# “If my husband leaves me, I will go home and suffer, so better cling to him and hide this thing”: The influence of gender on Option B+ prevention of mother-to-child transmission participation in Malawi and Uganda

**DOI:** 10.1371/journal.pone.0178298

**Published:** 2017-06-08

**Authors:** Valerie L. Flax, Jennifer Yourkavitch, Elialilia S. Okello, John Kadzandira, Anne Ruhweza Katahoire, Alister C. Munthali

**Affiliations:** 1 MEASURE Evaluation, Carolina Population Center, University of North Carolina, Chapel Hill, North Carolina, United States of America; 2 Department of Nutrition, Gillings School of Global Public Health, University of North Carolina, Chapel Hill, North Carolina, United States; 3 ICF, Fairfax, Virginia, United States of America; 4 Department of Psychiatry, Makerere University, Kampala, Uganda; 5 Centre for Child Health and Development, Makerere University, Kampala, Uganda; 6 Centre for Social Research, Chancellor College, University of Malawi, Zomba, Malawi; Tulane University School of Public Health and Tropical Medicine, UNITED STATES

## Abstract

The role of gender in prevention of mother-to-child transmission (PMTCT) participation under Option B+ has not been adequately studied, but it is critical for reducing losses to follow-up. This study used qualitative methods to examine the interplay of gender and individual, interpersonal, health system, and community factors that contribute to PMTCT participation in Malawi and Uganda. We conducted in-depth interviews with women in PMTCT, women lost to follow-up, government health workers, and stakeholders at organizations supporting PMTCT as well as focus group discussions with men. We analyzed the data using thematic content analysis. We found many similarities in key themes across respondent groups and between the two countries. The main facilitators of PMTCT participation were knowledge of the health benefits of ART, social support, and self-efficacy. The main barriers were fear of HIV disclosure and stigma and lack of social support, male involvement, self-efficacy, and agency. Under Option B+, women learn about their HIV status and start lifelong ART on the same day, before they have a chance to talk to their husbands or families. Respondents explained that very few husbands accompanied their wives to the clinic, because they felt it was a female space and were worried that others would think their wives were controlling them. Many respondents said women fear disclosing, because they fear HIV stigma as well as the risk of divorce and loss of economic support. If women do not disclose, it is difficult for them to participate in PMTCT in secret. If they do disclose, they must abide by their husbands’ decisions about their PMTCT participation, and some husbands are unsupportive or actively discouraging. To improve PMTCT participation, Ministries of Health should use evidence-based strategies to address HIV stigma, challenges related to disclosure, insufficient social support and male involvement, and underlying gender inequality.

## Introduction

In Malawi and Uganda, similar traditional gender norms guide daily life. Women’s roles center on marriage and motherhood, household duties, and caring for family members and the sick [1–3]. Women are supposed to consult with their husbands about household and health-related issues and to respect their husbands’ decisions [4]. Men’s roles are to lead and represent the family and to bring in income for household expenses [4, 5]. Men frequently have more than one wife or girlfriend, which is considered a sign of masculinity [6], whereas women are expected to be monogamous. These gender roles may constrain women’s access to health services, including their participation in the prevention of mother-to-child HIV transmission (PMTCT) program [7].

Globally, countries are working toward achieving the goal of having 90% of people diagnosed with HIV on sustained antiretroviral therapy (ART) [8]. This has been difficult to achieve in PMTCT programs in sub-Saharan Africa owing to weak health systems and a variety of individual, interpersonal, and cultural factors [9]. Enrollment in PMTCT has increased under Option B+ [10], which initiates all HIV-positive pregnant or breastfeeding women on lifelong ART at diagnosis [11], but estimates of losses to follow-up vary widely. In Malawi, 15% of women do not initiate ART and another 31% are lost to follow-up (LTFU) at 12 months [12, 13]. In Uganda, 16% of women do not initiate ART [14] and the proportion of women who are LTFU ranges from 8% to 70%, depending on the presence of programs to improve retention [15]. Studies that have examined barriers to PMTCT participation found that common constraints include fear of HIV stigma, fear of HIV disclosure, ART side effects, lack of funds for transport to a clinic, negative interactions with health workers, and lack of male involvement [3, 9, 16–24]. Most studies of barriers to PMTCT were conducted prior to the implementation of Option B+.

One important gap in knowledge about Option B+ programs is how gender interplays with individual, interpersonal, health system, and community factors that contribute to PMTCT participation. In addition, men’s perspectives on Option B+ are largely absent from national and global discussions. This study is the first to use qualitative research to assess gender-related facilitators and barriers to PMTCT participation under Option B+ in two countries. The findings are intended to inform policy discussions, program implementation, and related support for women in PMTCT care.

## Materials and methods

### Study overview

This study was conducted in 2015–2016 by MEASURE Evaluation in partnership with the Centre for Social Research, Chancellor College, University of Malawi and the Child Health and Development Centre, College of Health Sciences, Makerere University, Uganda. We used in-depth interviews and focus group discussions (FGDs) to obtain data from women participating in Option B+, women who were LTFU from Option B+, health workers who provide Option B+ services, stakeholders in organizations supporting those services, and men in the community. Data were collected in urban and rural areas of four districts in each country.

### Study sites

We chose to conduct this study in Malawi and Uganda because both countries have mature Option B+ PMTCT programs that are implemented nationwide. In 2011, Malawi became the first country to adopt Option B+, and Uganda adopted it in 2012. The study took place at government health facilities that provide free, comprehensive PMTCT (Malawi) or elimination of mother-to-child transmission (EMTCT; Uganda) services and in the surrounding communities. For simplicity, we use the term PMTCT henceforth for the programs in both countries.

We included nine facilities in the Central and Southern Regions of Malawi and eight facilities in the Central 1 and Southwestern Regions of Uganda. In Malawi, five urban facilities were located in Lilongwe and Blantyre, and four rural facilities were located in Dowa and Thyolo Districts. We intended to collect data at eight health facilities in Malawi, but one clinic in Lilongwe District was added because of challenges with contacting LTFU women at another clinic in that district. In Uganda, four urban facilities were located in Kampala and Mbarara and four rural facilities were located in Masaka and Ntungamo Districts. The selection of the study districts and health facilities was based on urban/rural location and history of PMTCT service delivery.

### Sample and eligibility

We used a purposive sampling method and chose our sample sizes in advance with the goal of having enough participants to attain saturation in subgroups we wanted to compare. Saturation is the point at which no new information or themes are observed in the data collected. A minimum of six interviews is needed to achieve saturation in qualitative research [25]. Our planned sample in each country consisted of 32 women participating in PMTCT (four per clinic), 32 women who were LTFU (four per clinic), 16 health workers (two per clinic), eight stakeholders (one per district), and eight FGDs (one per district) with approximately 10 men in each. Eligibility criteria for each type of participant and the actual numbers enrolled are shown in **Table 1**. We chose the types of participants included in this study to allow us to understand gender-related and other facilitators and barriers to Option B+ participation at the individual, interpersonal, health system, and community levels. We enrolled fewer LTFU women than planned in Uganda because of difficulties identifying sufficient numbers of those women at some clinics. In Malawi, we enrolled fewer stakeholders than expected because one district had no organizations supporting PMTCT services.

**Table 1:** Respondents, eligibility criteria, and data collection methods in a study of gender and Option B+ in Malawi and Uganda.

Type of study participant	Malawi enrollment	Uganda enrollment	Eligibility criteria	Data collection method
PMTCT women	32	32	• ≥ 18 years of age • HIV-positive • Current PMTCT participant • Pregnant or has child < 24 months of age	In-depth interviews
LTFU women	32	16	• ≥ 18 years of age • HIV-positive • Enrolled in PMTCT during current or most recent pregnancy • Has not been to the clinic to collect ART for > 60 days • Pregnant or has child < 24 months of age	In-depth interviews
Health workers	16	17	• ≥ 18 years of age • Provides PMTCT services at one of the study clinics	In-depth interviews
Stakeholders	6	8	• ≥ 18 years of age • Works for an organization that supports or provides PMTCT services	In-depth interviews
Men	8 groups, 77 participants	8 groups, 73 participants	• ≥ 20 years of age • Lives in the catchment area of one of the study clinics	Focus group discussions

### Data collection

Ten Malawian and nine Ugandan interviewers collected the data. In each country, the interviewers were divided into two teams that collected data simultaneously in different districts. Interviewers obtained signed or thumb-printed informed consent from all participants. The University of North Carolina Institutional Review Board (#15–1454), the Malawi Ministry of Health’s National Health Services Research Committee (#15/4/1416), the Makerere University School of Medicine Research and Ethics Committee (#2015–128), and the Uganda National Council for Science and Technology provided ethical approval for the study. All participants received approximately US$5 to cover their transportation costs.

Interviewers approached PMTCT women during a clinic visit, assessed them for eligibility, invited them to participate in the study, and interviewed them at the end of their clinic visit. Clinic staff members helped to identify LTFU women using records indicating that they had not returned to the clinic to collect ART for at least 60 days. Interviewers contacted LTFU women by cell phone or through community health workers and interviewed them at the location of their choice, in the community or at the clinic.

The interview guides for PMTCT women and LTFU women explored factors potentially related to participation, such as HIV disclosure, stigma and violence, distance to the clinic, side effects of treatment, experiences at the clinic, women’s workload, social support provided by family members, community perceptions of HIV-positive women, and male involvement in PMTCT programs. If they had a child < 24 months, we asked about their infant and young child feeding practices to date. With LTFU women, we probed about the factors that led them to drop out of the program. All question guides are included in Supporting Information files.

The officer in charge at each clinic identified health workers who provide PMTCT services; their interviews took place at health facilities. District health staff members provided information about stakeholder organizations in the district. Interviewers contacted stakeholders and interviewed them either at a health facility or at their project offices. In-depth interviews with health workers and stakeholders included similar types of questions and focused on their perceptions of the facilitators and barriers to PMTCT participation and their experiences with male involvement in PMTCT.

Village chiefs and local leaders helped recruit men for FGDs, which were conducted in the communities surrounding each health facility. We sampled men in general and did not specify that they had to have a certain HIV status or be in a relationship with an HIV-positive woman, because we wanted to learn about social norms in perceptions about HIV-positive women and PMTCT. The FGD question guide explored HIV disclosure, stigma and violence related to HIV, gender roles within families, male support for and involvement in PMTCT, ways of improving male participation in PMTCT, and community perceptions about HIV.

We field-tested data collection tools during interviewer training. We developed all consent and data collection forms in English and translated them into Chichewa in Malawi and into Luganda and Runyankole in Uganda. Interviewers digitally recorded in-depth interviews and FGDs, transcribed them verbatim in the local language, and translated them into English. We checked English transcripts for completeness and then uploaded them to Dedoose for analysis.

### Data analysis

Two research assistants and two investigators worked together to develop the codebooks for each type of participant. We initially used deductive codes based on the question guides and added inductive codes that emerged from the data. We independently coded one or two transcripts of each type of participant and compared our codes to help ensure consistency in code application and to finalize the codebook. The research assistants then coded the rest of the transcripts.

We met regularly to discuss the coding process and to identify key themes and codes that fit within the themes. The research assistants generated data matrices, following the method proposed by Miles & Huberman [26], to facilitate analysis and selection of quotations.

## Results

### Participant characteristics

The characteristics of women in PMTCT and women who were LTFU are shown in **Table 2**. On average, the women interviewed in Malawi were older, had higher parity and fewer household items, and reported food insecurity more often than those in Uganda. The Malawian women’s youngest children were slightly older on average than those of the Ugandan women. This is consistent with recommended breastfeeding duration for HIV-positive women in the two countries (24 months in Malawi and 12 months in Uganda) and, therefore, with the length of follow-up in the PMTCT program. A greater proportion of Ugandan than Malawian women in our sample were salaried workers and had husbands or partners who were on ART.

**Table 2:** Characteristics of women in PMTCT and those who were LTFU in a study of gender and Option B+ in Malawi and Uganda.

Characteristics	Malawi		Uganda	
	PMTCT women (n = 32)	LTFU women (n = 32)	PMTCT women (n = 32)	LTFU women (n = 16)
Age (years)	30.1	29.9	26.8	26.9
Years in PMTCT	3.5	1.3	2.8	1.6
Parity	3.6	3.7	2.3	2.9
Youngest child’s age (months)	11.7 ^a^	11.8^b^	7.8	9.9
Education (years)	5.1	7.5	7.4	6.8
Household items (max 10)	2.4	3.1	5.5	4.3
Marital status Married Separated/divorced Widowed Single/never married Missing	% (n) 84 (27) 9 (3) 6 (2) 0 (0) -	% (n) 72 (23) 9 (3) 9 (3) 9 (3) -	% (n) 75 (24) 15 (5) 3 (1) 0 (0) 3 (1)	% (n) 75 (12) 13 (2) 6 (1) 0 (0) 6 (1)
Employment Housewife Agricultural worker Petty trader Salaried worker Other	34 (11) 31 (10) 22 (7) 3 (1) 9 (3)	13 (4) 31 (10) 31 (10) 6 (2) 19 (6)	22 (7) 25 (8) 6 (2) 22 (7) 25 (8)	19 (3) 50 (8) 0 (0) 12 (2) 19 (3)
Husband/partner is HIV-positive Yes, on ART Yes, not on ART No Not tested/don’t know	15 (5) 47 (15) 19 (6) 19 (6)	17 (5)^c^ 14 (4) 10 (3) 59 (17)	37 (12) 13 (4) 28 (9) 22 (7)	25 (4) 19 (3) 25 (4) 31 (5)
Presence of household food insecurity	72 (23)	69 (22)	38 (12)	63 (10)
Frequency of food insecurity At least one day per week A few days per month Irregularly	(n = 23) 26 (6) 61 (14) 13 (3)	(n = 22) 54 (12) 32 (7) 14 (3)	(n = 12) 25 (3) 17 (2) 58 (7)	(n = 10) 20 (2) 30 (3) 50 (5)

^a^The sample for child’s age is (n = 27) because 5 women were pregnant.

^b^The sample for child’s age is (n = 30) because 2 women were pregnant.

^c^Three LTFU women were single, so the sample is n = 29.

The types of health workers included in the study were: nurses or nurse/midwives (n = 13) and health surveillance assistants (n = 3) in Malawi; and nurses or nurse/midwives (n = 15), clinical officer (n = 1), and peer mother (n = 1) in Uganda. They had been working in PMTCT for an average of 4.6 years in Malawi and 5.6 years in Uganda. Stakeholders worked for Mothers to Mothers (n = 3), Baylor College of Medicine (n = 2), or Management Sciences for Health (n = 1) in Malawi, and for health facilities (n = 2), non-governmental organizations (n = 3), Makerere University Joint AIDS Programme (n = 2), and the Infectious Disease Institute (n = 1) in Uganda. They had worked in their organizations for an average of 3.8 years in both countries. In Malawi and Uganda, respectively, men participating in FGDs were 33.2 and 37.6 years of age, on average, and had 7.5 and 9.8 years of education; 90 percent and 93 percent were married.

### Overview of findings

We found many similarities in the key themes across respondent groups and between the two countries (**Table 3**). The main facilitators of PMTCT participation in both countries were knowledge of the health benefits of taking ART; social support from family, friends, and health workers; and self-efficacy to make clinic visits and take ART regularly. The main barriers to participation were fear of HIV disclosure and stigma; lack of social support and male involvement; and lack of self-efficacy and agency. We describe each of these themes and indicate how they are influenced by gender norms. Differences in findings from the two countries are also noted.

**Table 3:** PMTCT facilitators and barriers by type of participant and country^a^.

	PMTCT women		LTFU women		Health workers		Stakeholders		Men	
	*Malawi*	*Uganda*	*Malawi*	*Uganda*	*Malawi*	*Uganda*	*Malawi*	*Uganda*	*Malawi*	*Uganda*
	(n = 32)	(n = 32)	(n = 32)	(n = 16)	(n = 16)	(n = 17)	(n = 6)	(n = 8)	(n = 8)	(n = 8)
Facilitators										
Improved health of woman	27	29	11	13	8	8	4	3	6	5
Prevent HIV transmission to child	20	19	22	10	11	14	4	5	7	6
Social support	29	32	9	6	12	8	4	5	6	5
Self-efficacy to participate	15	16	-	-	-	-	-	-	-	-
Barriers										
Fear of stigma/stigma experiences	24	29	25	26	15	14	5	8	7	8
Fear of disclosure/lack of disclosure	23	26	29	14	13	14	6	8	6	5
Lack of male involvement	20	16	15	10	11	14	5	5	4	6
Lack of self-efficacy to participate	14	11	13	3	1	3	-	-	-	-
Fear of divorce or loss of economic support	6	2	1	6	9	8	4	7	5	7
Lack of social support	2	9	1	6	-	-	-	-	-	-
Food insecurity/poverty	18	9	18	4	1	2	-	-	-	-
Lack of money for transport to clinic	-	10	3	4	1	8	-	4	-	-
Long wait time at clinic	3	13	2	2	-	-	-	-	-	-
Rude or disrespectful health workers	1	11	4	2	-	-	-	-	-	-
Physical, verbal, psychological abuse	1	10	5	7	2	8	1	5	3	4

^a^The number in each cell represents the number of participants who mentioned the issue, based on content analysis.

### Facilitators of PMTCT participation

#### Knowledge of health benefits

Women in Malawi and Uganda understood that following the PMTCT guidelines (i.e., taking ART daily and exclusively breastfeeding until six months) was beneficial for their own health and that of their children. Two women explained:

“The benefit is that your child grows stronger and healthier. You might have the infection, but your child will not get it from you. And when the child grows, he feels proud that you cared for him.”—Malawian PMTCT Woman #2

“I have good health and I look after my children…This program is life…[by stopping participation] you are looking for death.”—Ugandan PMTCT Woman #24

Not only knowledge but also experience of the health benefits encourages women to visit the clinic and follow the guidelines. According to a health worker in Uganda (#78), *“*The fact that HIV-positive women can have HIV-negative babies encourages women to participate.*”*

Maintaining maternal and child health is important because motherhood and caring for the family are key gender roles assigned to women. A healthy child is seen as a sign of a good mother. Women need to stay healthy themselves so that they can continue to be caregivers. Thus, PMTCT participation reinforces their gender roles. A few respondents mentioned that the health benefits for the child are also benefits for the mother, because traditional gender norms dictate that mothers are responsible for child care, and it is easier for them to care for a child who is not ill. An LTFU woman in Malawi (#39) explained, *“*Women benefit more because of the gender roles assigned to women. Because everything that happens [to the children] is the responsibility of women.*”*

#### Social support

Both PMTCT women and LTFU women described the types of social support they received and the importance of support in encouraging PMTCT participation. Nearly all the PMTCT women in both countries and about half the LTFU women in Uganda and two-thirds in Malawi said that they had family members, friends, health workers, or other HIV-positive women at the clinic who supported their participation in PMTCT. Here are some examples:

“He [my husband] reminds me to take my medication and to go to the clinic on time.”*—*Malawian PMTCT Woman #1

“The health workers continue encouraging you to adhere to treatment and advise you on how to live positively, and they share with you some important information that you may not know…It gives me pleasure and comfort when I sit with my health workers and chat.”—Ugandan PMTCT Woman #5

Women described social support as expressions of empathy, tangible aid, or advice and suggestions. They felt that social support was an important factor in encouraging them to go to the clinic, take their medication regularly, and not lose hope in their situation. The most common forms of support, usually from husbands/partners, were money for transport to the clinic and reminders to take ART.

#### Self-efficacy

Self-efficacy is defined as belief in one’s ability to engage in certain behaviors and to exert control over one’s own motivation and social environment [27]. Many of the PMTCT women in Malawi and Uganda described high levels of self-efficacy and they were motivated by the health and longevity benefits of participation in the program. Here are two examples:

“The feeling or the will to stay healthy is the one which enables me to participate in this program.”—Malawian PMTCT Woman #2

“I keep reminding myself to take the drugs because I have nobody to remind me. I remind myself because it is my life.”*—*Ugandan PMTCT Woman #14

Some women with high levels of self-efficacy said that they might not be able to participate in the program without financial or other support from their husbands or family members.

### Barriers to PMTCT participation

#### Fear of HIV disclosure

All types of respondents in both countries said that fear of disclosure was a major factor in women’s decision to stop PMTCT participation and in their experiences with PMTCT services. The current operational setup of Option B+, whereby women are tested for HIV during their first antenatal care visit, places the burden on the woman to disclose her status. In Malawi and Uganda, very few husbands accompany their wives to antenatal care or PMTCT visits. In most cases, the wife learns her status alone and then must decide whether, when, and to whom to disclose. If she discloses and her family is unaccepting, it is hard for her to stay in the program. Likewise, if she does not disclose her status, she does not get the support she needs to participate in the program. This is a no-win situation for some women.

Women especially fear disclosing to their husbands, because they are afraid they will be blamed for bringing HIV into the relationship. For example, an FGD participant in Uganda (#89) explained, “A woman finds it difficult to disclose to her husband because he can yell at her, talk to her rudely, [saying] that she is the one who gave him HIV.” In addition, most Malawian and Ugandan women are economically dependent on their husbands and afraid that they will be abandoned if their husbands find out they are HIV-positive. Wife and mother are their most important roles as women, and an HIV diagnosis threatens those roles with the risk of separation or divorce. For example, a health worker (#65) in Malawi explained, “It’s the men who are breadwinners, so [women] are afraid to tell them their status, because they fear being chased from the house or that their husband would leave them for another woman.”

Respondents in both countries described discordant status within a couple as a major problem for women but not for men. A health worker in Uganda (#73) stated, “If there is discordance and [the] woman is positive, then she is in trouble [from the husband], but if he is positive, then he may keep it a secret and continue his life.” Anticipation of a husband’s reaction, together with the belief that he is HIV-negative, contributes to women’s fear of disclosure.

Various types of respondents described the difficulties that women who decide not to disclose their HIV status face in staying in the PMTCT program. It is hard for them to take their ART daily without having others find out, and problematic for them to go to the clinic regularly to collect more ART. Here are some illustrative quotations:

“They [women who have not disclosed] are afraid of their husbands or the ones they are staying with. It becomes a challenge to take their medication, because they are afraid that they may get caught.”—Malawian Health Worker #71

“The fact that he [my husband] doesn’t know that I am HIV-positive is what stops me from participating in the PMTCT program.”—Ugandan LTFU Woman #49

The design of service provision at most of the health facilities made many women in this study worry about inadvertent disclosure to others in the community. This could occur because women in PMTCT are asked to go to a certain part of the facility or because the PMTCT clinic is on a specific day. The women might see people they know, and those people might figure out that they are HIV-positive. In other cases, a woman’s status may be disclosed through lack of privacy at the clinic or when health workers call out patients’ names in a public space. Two LTFU women explained:

“We should be attended to inside the clinic because we would like to keep our HIV status secret.”—Ugandan LTFU Woman #47

“I have always wanted to continue with the treatment, but I don’t like the long queues and the arrangement itself, whereby they give you one date for receiving ART and you are put in a separate queue from other patients with general illnesses. If it were one queue, then it would be much better, rather than calling out names of people going to receive ART. That is a burden to me.”—Malawian LTFU Woman #53

Despite all the issues related to HIV disclosure, many women in this study were able to disclose to someone close to them. According to the health workers and women in our study, women are advised to disclose to a relative or a close friend in case anything happens and they need someone to go to the clinic to collect ART. Women themselves know to whom they feel comfortable disclosing. A health worker in Malawi (#68) explained, “Everyone knows who can be trusted with such issues without humiliating them about their status.” All the PMTCT and some of the LTFU women had disclosed their status to at least one person. However, a third of LTFU women in Malawi and half of LTFU women in Uganda either had not disclosed to their husbands or had disclosed to no one. Of the PMTCT women and LTFU women who had disclosed to one or a few others, most had done so reluctantly, because they feared being stigmatized and gossiped about.

#### Fear of HIV stigma

Many respondents in both countries described the interrelationship of HIV disclosure and stigma. Women who were worried about stigma were also worried about disclosure, and some respondents described the stigma that still exists in their families and communities. Women spoke of hurtful, fatalistic attitudes toward HIV-positive people, and how stigmatizing gossip harmed them. A PMTCT participant in Uganda (#6) explained, “You find people busy talking about you and you also find yourself having low self-esteem.” Stigma in communities and health facilities also contributed to women dropping out of PMTCT. According to a health worker in Malawi (#75), “When they [women] face stigma in the communities, they find it hard to come to the hospital. And even here at the hospital, if we show that we don’t want to help them because of their status, then they feel the stigma and just decide to drop out.”

Respondents generally believed that women face more stigma than men. HIV stigma in our sample was related, in part, to community perceptions of HIV-positive women. Some respondents in both countries talked about people who assumed that women with HIV were promiscuous, prostitutes, or “already dead,” and therefore could not fulfill their role as good and faithful wives, as prescribed by local gender norms. For example:

“If a woman is HIV-positive, people think that she was a prostitute…and then these [women] are discriminated…when they are known to be on ART. As such, women are ashamed to participate in PMTCT or HIV testing, for fear of being shamed.”—Malawian FGD #97

“Of course women are more stigmatized than men. Because we have this mentality that it is okay for [men] to do whatever they want, like they can have many women and it is okay. But if you are a woman and you are a discordant woman, your husband is negative and you are positive, the whole village will think that you are a slut or something like that. And if it’s the other way round, and it’s the man who is HIV-positive and you are not, they will actually be telling you, please support your husband, take care of him, make sure this and that, but if you are a woman, most probably they will throw you out of the house. It affects women more than it would men.”—Ugandan Stakeholder #84

Respondents described inequality in the stigmatization of women and men. Women in both countries had very similar perceptions about why HIV-positive women are more stigmatized than men. For example, a Ugandan LTFU woman (#37) said, “They [community members] can’t gossip about a man the way they gossip about a woman. They don’t despise men with HIV the way they despise women with HIV. They fear men.”

Despite these perceptions, some women, especially some of those participating in PMTCT, did not experience stigma, and other respondents thought that it was less common than in earlier times. A PMTCT woman in Uganda (#25) said, “Currently, there are so many people who are HIV-positive, so we no longer have people who stigmatize others because they are HIV-positive.” Several respondents believed that there were no differences in how HIV-positive men and women were viewed in the community. A few thought that women simply reacted differently to stigma and gossip than men—a point of view that one woman explained as follows:

“There is no difference. It’s just that women react differently as compared to men. Usually men are stronger in handling issues. For example, if women heard that someone was talking about them, they would react in a certain way, whilst men would just brush it off. [It’s] not necessarily that women are facing different treatment than men.”—Malawian PMTCT Woman #6

#### Lack of social support and male involvement

Lack of social support and male involvement in PMTCT are intertwined. When male partners are not involved, women lose a major source of support and encouragement for participation in the program. Many PMTCT or LTFU women in this study believed they had enough support for PMTCT participation, but some LTFU women either had no support or had negligent or actively unsupportive family members, especially husbands. If a husband was angry or threatening, the woman had difficulty continuing with PMTCT. The words of a health worker illustrate this point:

“They [husbands] threaten them [their wives] because they don’t want their wives to come here [to the health facility]. They know that if their wives are seen coming here to PMTCT, then they will know that they are receiving ART. And they are ashamed that people would also think that they, too, are taking ART.”—Malawian Health Worker #72

In addition to threatening, some husbands made it difficult for their wives to go to the clinic or told them to stop taking their medication. For example, an LTFU woman in Uganda (#33) was married to a teacher with a regular salary, but he would not give her money for transportation to the clinic. She said, “He told me that he does not have money [for me] to go to the hospital for medication. He told me to go work for it, then I can go and get the medicine.” Husbands with multiple wives or partners were often not supportive or not around. In other cases, a husband withdrew support by leaving the marriage.

Both PMTCT and LTFU women talked about instability in their relationships with their husbands and partners and the lack of trust and communication within couples. In some cases, when a relationship was already unstable, it became worse when the woman disclosed her HIV status. In other cases, the disclosure brought on instability and led to the end of the relationship. For example:

“My husband, as I already said, he doesn’t stay long at home [because he has other wives]. At first, he encouraged me to go for VCT [voluntary HIV counseling and testing], but now we quarrel a lot. That’s all I can say.”—Malawian LTFU Woman #58

A lack of trust in relationships makes it difficult for women to disclose, which makes it hard for them to continue participating in the PMTCT program. Many men, too, are fearful of disclosing their HIV status to a spouse; often a woman learns her husband’s HIV status only after disclosing her status to him. A PMTCT woman in Uganda (#9) explained, “He was the first [to learn he was HIV-positive], but he didn’t tell me. He kept it a secret.”

Male participation in PMTCT is a form of social support for women’s participation. However, very few men accompany their wives to the clinic for PMTCT visits in either country. The policy at government clinics in Malawi and Uganda to encourage male participation—by allowing women who come with their husbands to the health facility for PMTCT services to go to the front of the queue—has not been successful. Men do not get involved because they want to maintain their reputation, and being seen going to the clinic with a woman is a sign that the woman is “wearing the trousers” in the family. Men in both countries described this point of view:

“I think it is very difficult for most men in this community to come out in the open and be involved in issues to do with HIV/AIDS…A lot of them are still quite fearful of being laughed at, and they want to maintain a good reputation. I may say that I have never seen a man and his wife going to the clinic together under this program.”—Malawian FGD #107

“Most men think that when a man accompanies his wife to the clinic, it means that the man is being controlled by his wife. I have children, but I only accompanied my wife once to the clinic in my entire life, because I viewed it as waste of time when I reached there.”—Ugandan FGD #91

Some men view the clinic as a female space and are embarrassed to go there. According to two participants in an FGD in Malawi (#127):

Participant #7: “Men are usually shy to stay in front or with a lot of pregnant women on antenatal care or PMTCT days.”

Participant #1: “Men are usually afraid that everyone would know that ‘this one is the one responsible for this pregnancy.’”

Some men in Uganda also said that pride may keep men away from the clinic, because if a woman does not have nice clothing, it indicates that the man is not providing for his wife.

Some urban women in Uganda felt that it was not particularly useful for men to participate. A PMTCT woman in Uganda (#3) said, “There is nothing much they do—maybe just encouraging you.” This opinion was not evident in the data from Malawi.

#### Lack of self-efficacy and agency

Lack of self-efficacy, which was described as “laziness” by some respondents, was often attributed to women’s failure to accept their HIV status or to internalized stigma. Here are two examples:

“To be frank, the medical personnel at our clinic really try their best. They always encourage us to adhere to medication, but it’s just us people are not willing to accept our status and follow the right procedures.”—Malawian LTFU Woman #57

“Somebody can hate oneself and decide to leave the medicine.”*—*Ugandan PMTCT Woman #19

Self-efficacy and agency are related concepts, but agency describes the ability to act or exert power rather than just a *belief* in one’s ability. Respondents explained that women lacked agency because the norm was for husbands to make decisions within the family, including decisions about women’s health care. Women’s lack of agency may affect their self-efficacy by constraining their belief in their own ability to make decisions. Here are examples of quotations about women’s lack of control and the impact on PMTCT participation:

“We have some women who were told by their husbands that if you want to start taking that ART, you should leave my house. That means the woman has no say, though she wanted to start taking the medication. There was this other incident where the man reached a point of throwing the woman’s ART into the toilet because he didn’t want his wife to be taking it.”*—*Malawian Stakeholder #86

“One of the gender issues is that men take the decisions in the homes, so they decide whether you go to the hospital or not. It is the men who control the finances, so if they don’t give the money to go to the clinic, then how are you going? It is the men who determine where you get your treatment from.”—Ugandan Stakeholder #88

Women who appear to be “lazy” or to lack motivation to participate in PMTCT may have little or no control over decisions about their health.

In addition to being limited by their husbands’ decisions, many women feel they have little control over whether their husbands have other wives or relationships with other women. Gender roles allow men to show their masculinity by having multiple sex partners, often outside marriage. Although this behavior is tolerated, it has an impact on their wives and other female partners. A Malawian LTFU woman (#48) explained, “Sometimes it happens that maybe your husband doesn’t live right and that can make you get it [HIV].” Women’s lack of agency in relationships sometimes makes them feel helpless, which affects both their self-efficacy and their ability to participate in PMTCT. Another LTFU woman in Malawi (#56) explained how her lack of control pushed her to drop out of the program: “The other reason I stopped taking my medication was that he was still coming here to sleep with me. I was taking my medication and protecting myself, but he wasn’t. So I felt like I wasn’t doing anything [by taking the drugs].”

#### Other barriers

Several other barriers to women’s PMTCT participation were mentioned by respondents and were the main reason some LTFU women dropped out of the program; they included lack of money for transport to the clinic, lack of respect from health workers, food insecurity, ART side effects, faith beliefs, and being too busy to participate. Here we focus on health system barriers related to gender—specifically women’s need to travel to be caregivers in their extended families and how their missed visits interact with their lack of agency. A Uganda LTFU woman (#35) explained, “I went to visit my family. They called us on the phone [to say] that my brother was on his deathbed…When I came back, my appointment day for drug refill had already passed.” Several PMTCT and LTFU women talked about missed appointments and either not being welcomed back into the system or being refused a transfer to another clinic. For example:

“When I went to Blantyre, it was difficult for me to continue taking the pills because I didn’t have any idea where to get them. After two months, I came back. So, I went to the hospital and I asked them to give me a transfer to start getting the drugs at [another] hospital, but they refused…Since then up to now I haven’t gone back to get the drugs.”—Malawian LTFU Woman #48

These quotations show that some women feel they do not have the agency to change how they are served at the clinic. They are afraid to push too hard when services they need are not readily offered or when they are treated poorly. This may be related to female submissiveness dictated by local gender norms.

### Differences between Malawi and Uganda findings

Although there were many similarities in our results from Malawi and Uganda, we also found some differences between the two countries. There were more differences in responses from urban and rural participants in Uganda than in Malawi, where urban/rural responses were quite similar. For example, rural respondents in Uganda emphasized fear of stigma, workload, failure to disclose to husbands, negative interactions with health workers, lack of money for transport to the clinic, and poverty, whereas urban respondents focused on fatalism about the lack of a cure for HIV and low self-esteem. More details about the urban/rural differences are provided in the country reports [28, 29].

We found that more women in Uganda than in Malawi described physical, verbal, and psychological abuse by their husbands or other family members as a result of their participation in the PMTCT program or their HIV status. An LTFU woman in Uganda (#35) said,

“When I would come back [from the clinic], I would sleep outside because I feared to be caned [beaten]…He [my husband] was rude and harsh, and he did not want me to go there. If I went anyway, I would face it rough.”

This kind of treatment led women, like this one, to drop out of the program.

The Malawian women in our sample described a greater depth of poverty and food insecurity than did the Ugandan women. This sometimes led them to stop taking their ART because they did not have enough food to follow health workers’ recommendations to eat before taking their medication. It also impacted their ability to follow the child feeding recommendations. A Malawian woman in PMTCT (#32) with an eight-month-old child said, “I have only introduced porridge [to my daughter], just because of my situation. I wish I could feed her balanced foods, but I don’t have the money.”

Another difference was the length of time women in the two countries breastfed their children. Most women in Uganda reported weaning at or before 12 months, whereas most women in Malawi planned to breastfeed until 24 months. These breastfeeding practices align with the differing recommendations in the two countries.

## Discussion

Our findings reflect many of the factors previously reported as facilitators of and barriers to PMTCT, both prior to the implementation of Option B+ and in the few studies that have been conducted under Option B+. Like our study, other research documents the importance of having an HIV-negative baby as a major motivation for women to participate in PMTCT programs [19, 21, 30]. Our findings are also consistent with other studies in terms of some of the barriers to PMTCT participation, including lack of support from husbands and fear of HIV disclosure and stigma [3, 16–19, 21, 24, 31–33]. Some issues that have been highlighted as major barriers to PMTCT participation, such as long waiting times at the clinic, negative treatment by health workers, cost of transport to the clinic, and side effects of ART [9, 16, 17, 19, 24, 31–35], were raised in our study, mainly by participants in Uganda, but were not described as the main issues by most respondents. This study adds to the literature by including a thorough investigation of how gender and gender norms are related to the key facilitators of and barriers to PMTCT participation and by bringing men’s perspectives to light. Our findings support the notion that the lower status of women in society contributes to the multifactorial challenges they experience at the individual, interpersonal, and institutional levels in relation to PMTCT participation.

Underlying women’s participation in PMTCT are gender norms that confer respect and recognition on women who are married and have children, take care of household duties, and defer to men [1–3]. These norms permeate Malawian and Ugandan society to the extent that women perceive themselves as incomplete without a male partner, regardless of the nature of the relationship they are in, and having children gives them more status while cementing that relationship. PMTCT preserves women’s role as mothers, but their relationships are threatened by the knowledge that they are HIV-positive, which has implications for their status in society and in their families.

Although male partner involvement in PMTCT can improve women’s participation in the program [36–38], we found little evidence of male partner participation in our study, despite the PMTCT program’s prioritization of services for couples over individual women. Men in our sample asserted that the PMTCT clinic is a female space and that they would feel uncomfortable there and would be concerned about their reputations if seen going there. In addition, the male gender role is to provide for the family, and work may leave men no time to accompany their wives to antenatal care. Similar barriers have been reported in a review of male involvement in PMTCT programs [39].

The lack of male involvement in PMTCT is important because it means that most couples are not tested together. Typically, women are tested for HIV during the initial antenatal visit unless they opt out, which very few do [40]. Under Option B+, they learn about their HIV status and start on lifelong ART before they have a chance to talk to their husbands, partners, or other family members, or even to think about the implications for their lives [17]. Having the test on their own puts women in an awkward position, because they risk abandonment and loss of economic support when they disclose their status to their husbands or partners. The PMTCT program is sometimes referred to by community members as “the divorce program,” owing to its frequently negative impact on social relations within the structure of prevailing gender norms [7].

Studies of HIV disclosure by pregnant and postpartum women report wide variations in the proportion of women who disclose [41]. Studies in Malawi and Uganda indicate that the majority of women in PMTCT disclose their status, most frequently to their husbands or partners rather than to other family members [42, 43]. Their decision to disclose is related to individual, partner, and household factors. Women find it easier to disclose when they have lower levels of internalized stigma, their partners have previously been tested, and they are financially independent [41]. A study in Uganda found that when women did not disclose, it was owing to a fear of abandonment and of being blamed for bringing HIV into the family [44]. This aligns with our findings that many women have difficulty disclosing because they fear stigma or abandonment and are financially dependent on their husbands. These reasons for nondisclosure are linked to the power differentials and gender norms identified in this study.

Although some respondents indicated that stigma is no longer an issue, the majority said that it is still a major problem and that it takes different forms, such as gossip and avoidance. Stigma affects men and women differently, according to their prescribed gender roles. Respondents in our sample said that women experience HIV stigma more strongly than men because of gender differences and their position in society. In this study, stigma was closely linked to lack of disclosure and to lack of male involvement.

Our study had a few limitations in both content and implementation. First, we chose to include men in the community to learn about gender norms related to HIV in general and about male perceptions of PMTCT, HIV disclosure, and male involvement. It would have been helpful to include men whose wives are HIV-positive in order to understand their experiences with their wives’ disclosure and PMTCT participation or lack thereof. We recommend that future research specifically target this group. Second, our study focused on pregnant women and women with children younger than 24 months, so we cannot reach conclusions about long-term or lifelong use of ART, the strategy used in Option B+. Future studies may take a longitudinal approach by following women who continue with ART after their children “graduate” to determine what factors influence their continued participation. A longitudinal study could also follow women who drop out of the program to learn if or when they return to care and what their reasons are for returning. Third, in terms of study implementation, we found it challenging to enroll enough LTFU women, especially in Uganda. We learned that some women who are LTFU have transferred their care to another site, so there is a need for a system that can better track PMTCT participants across clinics.

## Conclusions

Our research identified important gender-related considerations for PMTCT programs in Malawi and Uganda. The similarities in findings in the two countries show that some issues need to be addressed across countries; however, the differences we noted between urban and rural areas of Uganda and between Malawi and Uganda indicate that context should be taken into consideration. With this in mind, the Ministries of Health should use evidence-based strategies, adapted to their countries as needed, to strengthen or scale up facility and community efforts to address issues such as women’s lack of self-efficacy or agency, lack of social support, fear of stigma, fear of disclosure, lack of male involvement in PMTCT, and gender inequality. Some methods for achieving these changes are:

- Peer-to-peer programs to improve self-efficacy and increase social support [45];
- Community-based HIV-stigma-reduction programs [46–48];
- Couples counseling and tracing and testing programs that increase male involvement in PMTCT [38, 49]; and
- Programs that improve gender equality by increasing women’s agency and access to credit or economic opportunities [50].

## Supporting information

S1 File In-depth interview guide for women participating in PMTCT. (DOC)

S2 File In-depth interview guide for women who have dropped out of PMTCT. (DOCX)

S3 File In-depth interview guide for health workers. (DOCX)

S4 File In-depth interview guide for stakeholders. (DOCX)

S5 File Focus group discussion guide for men. (DOCX)
