# Supplementary material for: “If my husband leaves me, I will go home and suffer, so better cling to him and hide this thing”: The influence of gender on Option B+ prevention of mother-to-child transmission participation in Malawi and Uganda
Source: PLoS One. 2017 Jun 8;12(6):e0178298. doi: 10.1371/journal.pone.0178298 (PMC5464556; doi:10.1371/journal.pone.0178298)
Supplement: S2 File — (DOCX) [file pone.0178298.s002.docx]

**STUDY ON GENDER AND PMTCT ADHERENCE IN MALAWI AND UGANDA**

**In-depth interview guide for women who have stopped participating in PMTCT**

Thank you for agreeing to participate in this interview. I will now turn on the digital voice recorder.

1. **HIV diagnosis, disclosure, stigma, and ARV use**
2. How did you find out that you were HIV positive? (**Probe**: when, where)
3. With whom have you shared your test results? (**Probe:** Which people did you tell about your test result [E.g. husband, other relatives, and friends]? Why? How did they react?)
4. Who didn’t you want to know about your test results? Why?
5. How long did you take antiretroviral drugs?
6. Please describe your experiences with taking antiretroviral drugs. (**Probe:** How did the drugs make you feel? How regularly did you take them?)
7. What made you decide to stop taking antiretroviral drugs?

1. **PMTCT Services**
2. Please tell me about what happened during your typical visit to the clinic when you were collecting antiretroviral drugs or seeking other services related to your HIV/AIDS disease (**Probe:** Take me through the different stages you went through? How long did it take once you were there? Please tell me about your experiences with the heath workers here.)
3. A. What advice did the health workers give about taking your medication? How frequently did they give this advice?

B. What advice did the health workers give you about feeding your baby? How frequently did they give this advice?

C. What advice did the health workers give you about disclosing your HIV status? How frequently did they give this advice?

1. What helped or encouraged you participate in the PMTCT program? (**Probe:** Ask for factors at different levels – individual, family, community, clinic)
2. What challenges did you face to participating in the PMTCT program? (**Probe:** Factors at different levels – individual, family, community, and clinic).
3. In your opinion, how could these challenges be addressed to enable you return to care?
4. In the future, who would you like to help or support your participation in the PMTCT program? (**Probe:** Roles that different people e.g. husband, mother, sister, and friends could play to help her attend clinic, take her medication, and follow child feeding guidelines. Ask why she thinks the people she has mentioned are the right people to help her.)
5. **Male involvement in PMTCT**
6. How do men participate in PMTCT?
7. How could men be more involved in the PMTCT program?
8. **If participant lives with a husband or male partner, then ask the following:**
9. A. I would like to know your husband’s HIV status. Has your husband/partner been tested for HIV?

B. Do you know the results?

C. If he is HIV-positive, does he take ARVs?

D. How did he feel about you taking ARVs?

E. How did his feelings about you being on ARVs influence your participation in PMTCT?

1. Please describe the division of labor in your household between you and your husband/male partner.

(**Probe:**

- What tasks do you perform? (E.g. working for money, doing household work, and caring for sick relatives).
- What tasks does your husband/partner perform? (E.g. working for money, doing household work, and caring for sick relatives).
- How does your workload influence your participation in the PMTCT program?
- How does your husbands’ workload influence his ability to help you participate in the PMTCT program?)

1. In what ways did your husband/partner help you participate in the PMTCT program? (**Probe:** help you to adhere to medication, breastfeed and feed your child?) (**If no help or support)**: Why didn’t he help you?)
2. In what ways did your husband/partner contribute to your dropping out of the PMTCT program? (**Probe:** What was his contribution to your stopping ART? Your adherence to child feeding practices?)
3. Does your husband/partner have other wives or partners? **(If he does):** How did his obligations to these partners affect his ability to support your participation in PMTCT? Your adherence to medication? Your adherence to child feeding practices?
4. **If participant has a child < 24 months, then ask the following:**
5. How old is your youngest child (in months)?
6. Please describe your feeding practices with your youngest child to date:

(**Probe:**

- Breastfeeding practices (How long did you exclusively breast feed, when did you initiate complementary foods)
- Timing of starting other fluids
- Timing of starting solid foods
- Who decided about these practices?
- How does your husband/partner help you with child feeding?)

1. **Violence and stigma**
2. Have you ever experienced stigma because of your participation in the PMTCT program or because you have HIV? **(If yes):** Please tell me about those experiences?
3. Have you ever experienced violence because of your participation in the PMTCT program or because you have HIV? (**Probe:** verbal abuse, physical violence) **(If yes):** Please tell me about those experiences.
4. Please describe opinions in this community about women with HIV.

(**Probe:**

- How do opinions about women with HIV differ from those about men with HIV?
- How do the opinions about HIV-positive women affect your participation in PMTCT services?)

1. **Programmatic/system elements**
2. How does the PMTCT program assist women with disclosing their HIV status to family members?
3. A. What at the health facility makes it difficult for women to stay in the PMTCT program?

B. What makes it difficult for women to adhere to medication?

C. What makes it difficult for women to adhere to the infant and young child feeding recommendations?

1. A. What are the benefits of staying in the PMTCT program?

B. What are the risks of staying in the PMTCT program?

1. A. What would be the benefits of stopping participation in the PMTCT program?

B. What are the risks of stopping participation in the PMTCT program?)

**SOCIOECONOMIC INFORMATION**

| **No.** | **QUESTIONS AND FILTERS** | **CODING CATEGORIES** | **SKIP INSTRUCTIONS** |
| --- | --- | --- | --- |
| 1 | How old are you? | Age in completed years: ____ |  |
| 2 | How many children have you given birth to? | Number of children: |  |
| 3 | How many of your (living) children are less than five years old? | Number of children < 5 years old: ____ ____ |  |
| 4 | What is your marital status? | Married…………………….…1  Separated/Divorced…………2  Widowed……………………..3  Single/never married……….4 | *SKIP to Q7*  *SKIP to Q7*  *SKIP to Q7* |
| 5 | Does your husband have other wives? | Yes…………………………1  No…………………………..0  Don’t know…………………8 | *SKIP to Q7*  *SKIP to Q7* |
| 6 | In total, including yourself, how many wives does your husband have? | One…………………………1  Two…………………………2  Three………………………..3  Four or more………………..4 |  |
| 7 | What is your highest level of education? (Compute: How many years of school completed) | Number of years of school completed: ____ ____ |  |
| 8 | What is your occupation (what kind of work do you mainly do)? | Housewife…………………….1  Agricultural work……………..2  Petty trader……………………3  Other………………………….4  *Specify:*_________________ |  |
| 9 | Are you paid in cash or kind for the work you do or are you not paid at all? | Cash only…………………..1  Cash and kind………………2  In kind only…………………3  Not paid…………………….4 |  |
| 10 | Are there times when you don’t have food in your household? | Yes…………………………1  No………………………….0 | *SKIP to Q12* |
| 11 | How frequently is your household without food? | At least one day per week……1  A few days per month…..….…2  Only during the rainy season…3  Only during dry season……….4  Other…………………………….5  *Specify:*________________ |  |

| **No.** | **QUESTIONS AND FILTERS** | **CODING CATEGORIES** | **SKIP INSTRUCTIONS** |
| --- | --- | --- | --- |
| 12 | Does your household have any of the following items? *(Multiple answers allowed)*   1. Electricity 2. Koloboyi/tadooba 3. Paraffin lamp other than a koloboyi/tadooba 4. Radio/tape player/other music player 5. Television 6. Mobile telephone 7. Foam/spring mattress 8. Sofa set 9. Table and chairs 10. Refrigerator | Yes = 1 No = 0  Yes = 1 No = 0  Yes = 1 No = 0  Yes = 1 No = 0  Yes = 1 No = 0  Yes = 1 No = 0  Yes = 1 No = 0  Yes = 1 No = 0  Yes = 1 No = 0  Yes = 1 No = 0 |  |
| 13 | How long does it take you to get from your home to the clinic where you usually receive PMTCT services? | < 1 hour ……………. 1  1-2 hours…………… 2  3-4 hours…………… 3  5 or more hours……. 4 |  |

**Thank you for your participation in this interview.**
