# Supplementary material for: “If my husband leaves me, I will go home and suffer, so better cling to him and hide this thing”: The influence of gender on Option B+ prevention of mother-to-child transmission participation in Malawi and Uganda
Source: PLoS One. 2017 Jun 8;12(6):e0178298. doi: 10.1371/journal.pone.0178298 (PMC5464556; doi:10.1371/journal.pone.0178298)
Supplement: S3 File — (DOCX) [file pone.0178298.s003.docx]

**STUDY ON GENDER AND PMTCT ADHERENCE IN UGANDA**

**In-depth interview guide for health workers providing PMTCT services**

Thank you for agreeing to participate in this interview. I will now turn on the digital voice recorder.

**A. HIV diagnosis, disclosure, and ARV use:**

1. a) To whom do women typically disclose their HIV status? Why?

b) From whom do they typically withhold that information? Why?

c) What would make it easier for a woman to disclose her status to her husband?

1. Please describe a typical PMTCT appointment. Begin with the woman’s arrival at the clinic and walk through each step, including who talks to her, where, what they talk about, and for how long. How long is this visit supposed to take?
2. During PMTCT visits,

a) What advice do you give about taking medication?

b) What advice do you give about feeding babies?

c) What advice do you give about disclosing HIV status?

d) What other information or advice do you give?

1. In your opinion,

a) How much do women in PMTCT adhere to regular use of ART?

b) What are the main reasons some women don’t take ART regularly?

1. In your opinion,

a) How much do women in PMTCT adhere to infant and young child feeding guidelines? (Probe: exclusive breastfeeding up to 6 months and continued breastfeeding to 24 months)

b) How do HIV-positive women decide when to stop exclusive breastfeeding? Why?

c) How do they decide when to completely wean the child?

1. What are your experiences with women’s participation in Option B+?
2. What enables women to stay on ART?
3. After enrolling in PMTCT, how long do they stay in the program? (Probe: do most stay in it throughout their pregnancy, through child’s first year, through child’s second year)
4. What are the main reasons women drop out of PMTCT? (Probe: distance to clinic, way they are treated at clinic, medication side effects, issues related to disclosure, husband’s refusal to accept wife’s HIV status, lack of support by husband, violence by husband, woman’s workload, food insecurity)
5. Of the women who drop out of PMTCT, what are factors that make them return to care?

**B. Male involvement**

- - - 1. How are husbands involved in PMTCT?
      2. How could men be more involved in PMTCT?

**C. Stigma and violence**

1. What kind of stigma do women experience because they are HIV-positive? What impact does stigma have on their PMTCT participation?
2. How does the stigma faced by HIV-positive women differ from that faced by HIV-positive men? Why?
3. What forms of violence do women experience because they have HIV? (Probe: verbal abuse, physical violence) From whom? How does this affect their PMTCT participation?
4. What do you do if women tell you about violence they have experienced?

**D. Programmatic/system elements:**

1. What changes to the PMTCT program would make it easier for women to continue participating?
2. How has Option B+ changed PMTCT program implementation?
3. What is your opinion about the Option B+ program? (Probe for both positive and negative aspects of the program for women and the country)
4. What are the challenges Option B+ has brought? How can these be addressed? By whom?

**E. Socioeconomic information**

| No. | QUESTIONS AND FILTERS | CODING CATEGORIES |
| --- | --- | --- |
| 1 | How old are you? | Age in completed years ____ ____ |
| 2 | How long have you been working in the PMTCT program? | Number of years  ____ ____ |
| 3 | Sex | Male……………………………..0  Female………………………….1 |
| 4 | Type of health worker | Specify the type: ________________ ______________________________ |

**Thank you for your participation in this interview**.
