# Supplementary material for: “If my husband leaves me, I will go home and suffer, so better cling to him and hide this thing”: The influence of gender on Option B+ prevention of mother-to-child transmission participation in Malawi and Uganda
Source: PLoS One. 2017 Jun 8;12(6):e0178298. doi: 10.1371/journal.pone.0178298 (PMC5464556; doi:10.1371/journal.pone.0178298)
Supplement: S4 File — (DOCX) [file pone.0178298.s004.docx]

**STUDY ON GENDER AND PMTCT ADHERENCE IN MALAWI AND UGANDA**

**In-depth interview guide for stakeholders**

Thank you for agreeing to participate in this interview. I will now turn on the digital voice recorder.

1. **HIV diagnosis, disclosure, and ARV use:**
2. To whom do women typically disclose their HIV status? Why?
3. From whom do they typically withhold that information? Why?
4. What would make it easier for women to disclose their status to their husband or male partner?
5. After getting their test results, when do most eligible women enroll in an PMTCT program?
6. After enrolling, how long do they stay in the program (**Probe:** Do most stay in it throughout the pregnancy, through child’s first year, through child’s second year)?
7. What are some of the issues related to women’s adherence to ART medication protocols?
8. How might a woman’s workload affect her participation in the PMTCT program? (**Probe:** Adherence to medication? Feeding her child according to recommendations?
9. What kind of gender issues prevent women from participating in the PMTCT program?
10. **Male involvement**
    - - 1. How are male partners involved in PMTCT? What are your thoughts regarding male involvement in PMTCT?
        2. How could men be more involved in PMTCT?
        3. How do male partners support women’s participation in the PMTCT program? How do male partners support women’s adherence to ART?
11. **Stigma and violence:**
12. What kind of stigma do women experience because they are HIV-positive? What impact does that have on their PMTCT participation?
13. How does the stigma faced by HIV-positive women differ from that faced by HIV-positive men? Why?
14. What forms of violence do women experience violence because they have HIV? From whom? How does this affect their PMTCT participation?

1. **Programmatic/system elements:**
2. What changes to the PMTCT program would make it easier for women to continue participating?
3. How has Option B+ changed PMTCT program implementation?
4. What is your opinion about the Option B+ program? (Probe for both positive and negative aspects of the program for women and the country)
5. What are the challenges Option B+ has brought? How can these be addressed. By whom?
6. **Socioeconomic information**
7. How old are you?
8. For how many years have you been working in this organization?

**Thank you for your participation in this interview**
