# Supplementary material for: “If my husband leaves me, I will go home and suffer, so better cling to him and hide this thing”: The influence of gender on Option B+ prevention of mother-to-child transmission participation in Malawi and Uganda
Source: PLoS One. 2017 Jun 8;12(6):e0178298. doi: 10.1371/journal.pone.0178298 (PMC5464556; doi:10.1371/journal.pone.0178298)
Supplement: S5 File — (DOCX) [file pone.0178298.s005.docx]

**STUDY ON GENDER AND PMTCT ADHERENCE IN MALAWI AND UGANDA**

**Focus group discussion guide for men living in catchment areas of PMTCT sites**

Thank you for agreeing to participate in this focus group discussion. I will now turn on the digital voice recorder.

**A. Knowledge of PMTCT**

1. Tell us what you know about PMTCT. (**Probe:** What is PMTCT? What happens in PMTCT? Who participates in the program?)

1. **Male involvement**
2. How are men in this community involved in the PMTCT program?
3. What kind of support can men in this community give to women participating in the PMTCT program? (**Probe**: support for their clinic visits, adherence to medication, and adherence to recommended child feeding practices)
4. What could be done to make men more involved in the PMTCT program?
5. **HIV diagnosis, disclosure, and ARV use**
6. A. If a woman is pregnant and finds out she is HIV-positive, to whom would she typically disclose her HIV status?

B. From whom would she typically withhold that information? Why?

C. What would make it easier for women to disclose their status to their husbands?

1. In your opinion, how is a husband likely to react to their wife’s HIV disclosure?
2. How might this affect the woman’s participation in the PMTCT program?
3. What have you heard about HIV-positive women’s experiences participating in the PMTCT program? (**Probe:** at the clinic, in the community, at the individual level)
4. Under the current PMTCT program, women are enrolled for life. In your opinion:

A. What are some of the reasons the women in this community stay in the program?

B. What are some of the reasons that women in this community might stop participating in the PMTCT program? (**Probe:** treatment by clinic staff, distance to clinic, woman’s workload, ART side effects, family obligations)

1. What should be a husband’s role in helping or supporting his wife to participate in the PMTCT program? (**Probe:** support for clinic visits, ART adherence, adherence to recommended child feeding practices)
2. If a man is not HIV-positive but his wife is, how would this situation affect the support he offers to his wife to participate in the PMTCT program?
3. If a man has HIV but is not taking ART, what would be his most likely reaction or attitude about his female partner taking ART?
4. How does the husband’s reaction or attitude influence a woman’s participation in the PMTCT program?
5. **Stigma and violence**
6. A. What do people think about women with HIV in this community?

B. How do they treat them?

C. How do opinions about women with HIV differ from those about men with HIV?

D. How do opinions about HIV-positive women affect women’s participation in the PMTCT program?

E. How do opinions of community members about wives with HIV affect a husbands’ ability to support his wife’s participation in the PMTCT program? (**Probe** about HIV-related stigma, if necessary)

1. Tell us about violence against HIV-positive women.

A. What types of violence do HIV-positive women experience in this community? (**Probe:** verbal abuse, physical violence)

B. How does this violence affect women’s participation in the PMTCT program?

1. **Programmatic/system elements**
2. What changes to the PMTCT program would make it easier for women to continue participating in the program?
3. What is your view about husbands accompanying their wives to PMTCT clinic visits?
4. Why is it difficult for most husbands to accompany their wives to the PMTCT clinic?
5. What would make it easier for husbands to participate in wives’ PMTCT clinic visits?
6. What have you heard about the benefits of the PMTCT program?
7. What are the negative aspects of the PMTCT program?

**SOCIO-ECONOMIC INFORMATION**

| **Participant #** | **Age** | **Occupation** | **Education** | **Married (yes/no)** |
| --- | --- | --- | --- | --- |
| 1 |  |  |  |  |
| 2 |  |  |  |  |
| 3 |  |  |  |  |
| 4 |  |  |  |  |
| 5 |  |  |  |  |
| 6 |  |  |  |  |
| 7 |  |  |  |  |
| 8 |  |  |  |  |
| 9 |  |  |  |  |
| 10 |  |  |  |  |

**Thank you for your participation in the discussion**
